# Giant Ovarian Cystadenoma Mimicking Massive Ascites: A Case Report

**DOI:** 10.7759/cureus.117383

**Published:** 2026-10-03

**Authors:** Fabienne Lorenz, Peter Schaub, Rugile Razmiene, Sara Parolari, Jan Chmielewski, Christine Manser, Markus K Müller, Pascal Probst

**Affiliations:** 1 Department of Internal Medicine, Cantonal Hospital Thurgau, Frauenfeld, CHE; 2 Department of Gastroenterology, Cantonal Hospital Thurgau, Frauenfeld, CHE; 3 Department of Obstetrics and Gynaecology, Cantonal Hospital Thurgau, Frauenfeld, CHE; 4 Department of Surgery, Cantonal Hospital Thurgau, Frauenfeld, CHE

**Keywords:** cystadenoma, diagnostic laparoscopy, giant ovarian cyst, interdisciplinary management, presumed ascites

## Abstract

Giant ovarian cysts may present with nonspecific abdominal symptoms and can clinically and sonographically mimic massive ascites. This diagnostic overlap may lead to extensive workup for hepatic, cardiac, infectious, vascular, or malignant causes before the underlying gynecological pathology is recognized.

We report the case of a 24-year-old woman who presented with progressive abdominal distension, flatulence, and nonspecific abdominal discomfort. The initial ultrasound was interpreted as massive ascites, and 12 liters of presumed ascitic fluid were drained. Fluid analysis showed a very low cell count and no evidence of malignancy or infection. Further investigations, including blood tests, computed tomography (CT), and transjugular liver biopsy, did not reveal an underlying cause. During follow-up, recurrent intra-abdominal fluid was noted, later appearing loculated rather than freely distributed. After interdisciplinary discussion, diagnostic laparoscopy was performed and revealed a giant ovarian cyst measuring up to 18 cm in maximum diameter and containing approximately 2 liters of residual fluid. The patient underwent left-sided oophorectomy and histopathology showed a benign serous cystadenoma. The postoperative course was uneventful, and the patient remained symptom-free during follow-up.

This case highlights that giant ovarian cysts may be misinterpreted as ascites. In patients with presumed ascites of unclear origin and repeatedly negative diagnostic findings, cystic gynecological lesions should be considered. Diagnostic laparoscopy may be decisive when noninvasive investigations fail to establish the diagnosis and can allow simultaneous therapeutic intervention.

## Introduction

Ascites and ovarian cysts are distinct clinical entities that may both present with abdominal distension and nonspecific abdominal symptoms. Ascites is defined as the abnormal accumulation of fluid within the peritoneal cavity and is most commonly associated with chronic liver disease, but may also occur in cardiac, malignant, infectious, inflammatory, or vascular conditions [1,2]. Diagnostic evaluation usually includes clinical examination, ultrasound imaging, laboratory testing, cross-sectional imaging, and, when appropriate, analysis of ascitic fluid.

Ovarian cysts are common gynecological findings, particularly in women of reproductive age, and are benign and asymptomatic in most cases [3,4]. Functional ovarian cysts may resolve spontaneously, whereas larger or pathological cysts can cause pelvic pain, abdominal distension, menstrual irregularities, or complications such as torsion or rupture [3-5]. Giant ovarian cysts, defined as cysts with diameters greater than 10 cm, are rare in industrialized countries, as most adnexal lesions are diagnosed earlier due to the widespread availability of modern imaging [5,6]. The risk for malignancy is strongly age-dependent, being considerably lower in premenopausal women and increasing significantly after menopause; however, tumor markers may be nonspecific and do not necessarily determine the therapeutic strategy [6,7].

Although ascites and ovarian cysts are usually distinguishable by clinical and imaging findings, very large cystic ovarian lesions may clinically and sonographically resemble massive ascites. This diagnostic overlap can lead to extensive workup for presumed ascites before the underlying gynecological pathology is recognized [6]. Several sonographic and cross-sectional imaging features may aid in differentiating ascites from a cystic lesion. In particular, a definable wall or capsule may be identified in cystic lesions. In contrast to free intraperitoneal fluid, the bowel loops are not freely floating within the fluid but are displaced by the mass effect of the cystic lesion. In such cases, the typical intraperitoneal recesses should also be systematically assessed. Free ascites typically accumulates within these dependent spaces, whereas a cystic lesion remains anatomically confined to its location of origin and does not conform to the expected distribution of free intraperitoneal fluid. A particular diagnostic challenge is determining the organ of origin when a cystic lesion extends beyond the pelvis and occupies a substantial portion of the abdominal cavity [8].

We report the case of a young woman with progressive abdominal distension and presumed recurrent ascites, in whom repeated diagnostic investigations, such as repeated cross-sectional imaging, serial fluid aspirations, extensive laboratory investigations, and ultimately a liver biopsy, failed to identify a hepatic, vascular, infectious, or malignant cause. The particular diagnostic challenge in this case was that the cystic lesion remained undetected throughout the diagnostic work-up until the final stage of evaluation.

In the setting of an undiagnosed cystic abdominal lesion, thorough evaluation for potential malignancy is essential before considering any percutaneous aspiration or biopsy. This is particularly important because, in the event of an underlying malignant neoplasm, percutaneous intervention may carry a risk of tumor-cell seeding along the needle tract or into the peritoneal cavity. The imaging characteristics of the lesion should therefore be carefully assessed for features suggestive of malignancy such as multilocular lesions with diverse, inner solid masses/nodules [5,9], and appropriate tumor markers should be obtained, where clinically indicated, prior to any invasive procedure. In our case, however, the initial working diagnosis was unexplained ascites. Given the patient’s very young age, otherwise good health, and absence of a relevant family history of malignancy, diagnostic paracentesis was subsequently performed as part of further workup of the abdominal fluid. In the end, diagnostic laparoscopy revealed a giant benign ovarian cystadenoma.

## Case presentation

We report the case of a 24-year-old woman who presented to her general practitioner with a several-month history of persistent flatulence, progressive abdominal distension, and nonspecific abdominal discomfort. There were no menstrual irregularities present, but the patient reported intentional weight loss of up to 40 kg over a period of 2 years, unrelated to the abdominal distension. She had no relevant medical and surgical history. There was no family history of malignancy. The initial ultrasound was interpreted as massive ascites, as shown in Figures 1-2, because there was no cyst wall definable. A retrospective review of the initial ultrasound images revealed that the bowel loops were not freely floating within the fluid, but rather displaced by the underlying lesion. However, in the absence of a clearly delineated cystic structure or identifiable cyst wall, the findings were initially interpreted as ascites.

**Figure 1:**
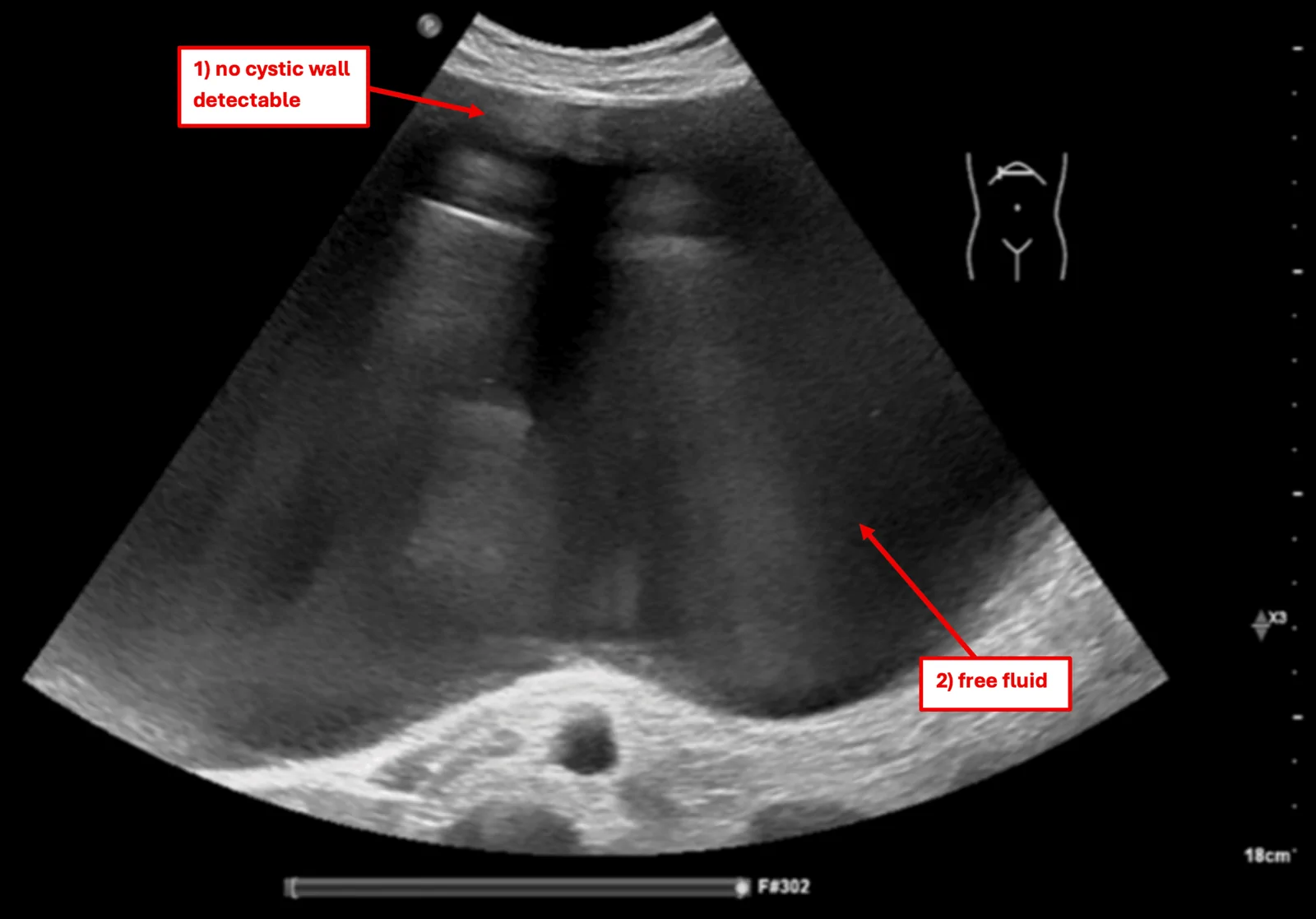
Abdominal ultrasound initially interpreted as ascites 1) No cyst wall detectable; 2) Free fluid, no freely floating bowel loops present, other organs seem rather displaced

**Figure 2:**
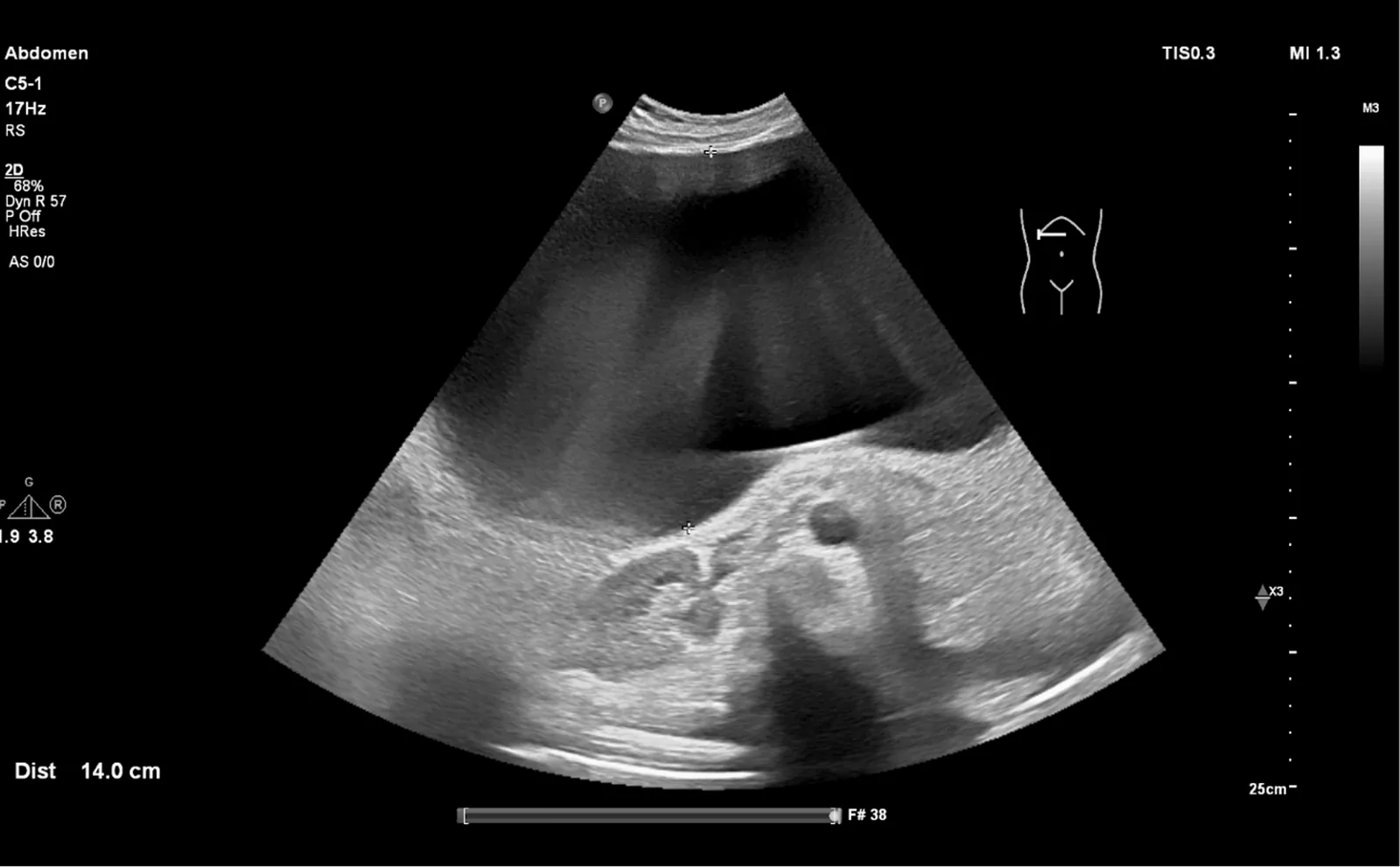
Abdominal ultrasound showing an intra-abdominal fluid collection

Due to the patient’s nonspecific symptoms and the unclear origin of the presumed ascites, the patient was referred to the gastroenterology department for further diagnostic evaluation, including ultrasound-guided puncture.

During the initial ultrasound-guided puncture of the presumed ascitic fluid, 12 liters of fluid were drained from the abdomen. Fluid analysis, including cytological examination, showed a very low cell count and no evidence of infection or malignancy; in retrospect, the fluid analysis could have suggested a cystic lesion as the origin of the fluid. Further blood tests showed no evidence of hepatic disease or infection (Table 1). The serum-ascites albumin gradient (SAAG) was >4.11 g/dL, based on a serum albumin concentration of 44.1 g/L and an ascitic fluid albumin concentration of <3 g/L. This value is significantly above the threshold of 1.1 g/dL, which is typically indicative of a portal hypertensive etiology. No further cystic fluid markers were assessed in the aspirated fluid, as the working diagnosis at that time was ascites of unclear etiology rather than a cystic lesion.

**Table 1:** Laboratory analysis results The initial serum–ascites albumin gradient (SAAG) was >4.11 g/dL, based on a serum albumin concentration of 44.1 g/L and an ascitic fluid albumin concentration of <3 g/L. The second SAAG was 42.7 − 11 = 31.7 g/L = 3.17 g/dL. Ascitic fluid analysis revealed a very low leucocyte count with an absolute polymorphonuclear cell count of approximately 6-7 cells/µL, without evidence of neutrophilic inflammation. eGFR: estimated glomerular filtration rate; CKD-EPI: Chronic Kidney Disease Epidemiology Collaboration; LDH: lactate dehydrogenase; AST: aspartate transferase; ALT: alanine aminotransferase; GGT: gamma-glutamyl transferase; ALP: alkaline phosphatase; TSH: thyroid-stimulating hormone; HBs: hepatitis B surface; HCV: hepatitis C virus; hCG: human chorionic gonadotropin; ANA: antinuclear antibody

Date	Category	Parameter	Unit	Reference range	Result
Day 25	Clinical chemistry	Sodium	mmol/L	136–145	138
Day 25	Clinical chemistry	Potassium	mmol/L	3.4–5.0	4
Day 25	Clinical chemistry	Creatinine	µmol/L	44–80	69
Day 25	Clinical chemistry	eGFR CKD-EPI	mL/min/1.73 m²	≥90	107
Day 25	Clinical chemistry	C-reactive protein	mg/L	<5	1
Day 25	Clinical chemistry	Total bilirubin	µmol/L	≤17	11
Day 25	Clinical chemistry	LDH	U/L	135–250	197
Day 25	Clinical chemistry	AST	U/L	10–50	27
Day 25	Clinical chemistry	ALT	U/L	10–50	17
Day 25	Clinical chemistry	GGT	U/L	<39	15
Day 25	Clinical chemistry	ALP	U/L	35–104	84
Day 25	Clinical chemistry	Total protein	g/L	62–80	75
Day 25	Clinical chemistry	Albumin	g/L	35–52	44.1
Day 25	Clinical chemistry	HbA1c (NGSP)	%	≤6.0	5.7
Day 25	Clinical chemistry	IgG	g/L	6.90–14.0	12.8
Day 25	Clinical chemistry	IgM	g/L	0.3–2.1	0.66
Day 25	Clinical chemistry	Ferritin	µg/L	10–150	61
Day 25	Clinical chemistry	TSH	mU/L	0.27–4.20	3.36
Day 25	Hematology	Hemoglobin	g/L	120–160	132
Day 25	Hematology	Leukocytes	10^9/L	4–10.5	6.3
Day 25	Hematology	Platelets	10^9/L	150–375	254
Day 25	Coagulation	Thromboplastin time	%	>70	89
Day 25	Coagulation	INR	-	<1.30	1.08
Day 25	Ascitic fluid	Fluid appearance		-	Clear
Day 25	Ascitic fluid	Fluid color	-	-	Colorless
Day 25	Ascitic fluid	Total cell count	/µL	-	18
Day 25	Ascitic fluid	Leukocytes	/µL	-	18
Day 25	Ascitic fluid	Polynuclear cells	%	-	33.3
Day 25	Ascitic fluid	Mononuclear cells	%	-	66.7
Day 25	Ascitic fluid	Erythrocytes	10^3/µL	-	0
Day 25	Ascitic fluid	LDH	U/L	-	<10
Day 25	Ascitic fluid	Protein	g/L	-	<3
Day 25	Ascitic fluid	Albumin	g/L	-	<3
Day 25	Ascitic fluid	Triglycerides	mmol/L	-	<0.1
Day 25	Ascitic fluid	Glucose	mmol/L	-	<0.1
Day 25	Ascitic fluid	Creatinine	µmol/L	-	<15
Day 25	Serology	HBs antigen	-	-	Negative
Day 25	Serology	Hepatitis B core antibody		-	Negative
Day 25	Serology	HBs antibody	U/L	-	<10
Day 25	Serology	HCV antibody		-	Negative
Day 25	Serology	HIV-1/2 antibody + p24 antigen			Negative
Day 33	Clinical chemistry	hCG	U/L	<5.0	40217
Day 33	Hematology	Hemoglobin	g/L	120–160	133
Day 33	Hematology	Leukocytes	10^9/L	4–10.5	8.3
Day 33	Hematology	Platelets	10^9/L	150–375	338
Day 33	Coagulation	Thromboplastin time	%	>70	94
Day 33	Coagulation	INR	-	<1.30	1.04
Day 33	Serology	ANA	Titer	<1:320	Negative
Day 33	Serology	ANA cytoplasmic pattern	Titer	<1:320	Negative
Day 33	Other	Iron in liver biopsy	Hepatic Iron Index	<1	0.4
Month 6	Clinical chemistry	Creatinine	µmol/L	44–80	65
Month 6	Clinical chemistry	eGFR CKD-EPI	mL/min/1.73 m²	≥90	115
Month 6	Clinical chemistry	Total bilirubin	µmol/L	≤17	12
Month 6	Clinical chemistry	AST	U/L	10–50	20
​​​​​​​Month 6	Clinical chemistry	ALT	U/L	10–50	15
​​​​​​​Month 6	Clinical chemistry	GGT	U/L	<39	9
​​​​​​​Month 6	Clinical chemistry	ALP	U/L	35–104	84
​​​​​​​Month 6	Clinical chemistry	Total protein	g/L	62–80	69
​​​​​​​Month 6	Clinical chemistry	Albumin	g/L	35–52	42.7
​​​​​​​Month 6	Clinical chemistry	TSH	mU/L	0.27–4.20	2.63
​​​​​​​Month 6	Hematology	Hemoglobin	g/L	120–160	132
​​​​​​​Month 6	Hematology	Leukocytes	10^9/L	4–10.5	6.8
​​​​​​​Month 6	Hematology	Platelets	10^9/L	150–375	255
​​​​​​​Month 6	Coagulation	Thromboplastin time	%	>70	89
​​​​​​​Month 6	Coagulation	INR	-	<1.30	1.08
​​​​​​​Month 6	Ascitic fluid	Fluid source	-	-	Ascites
​​​​​​​Month 6	Ascitic fluid	Fluid appearance		-	Clear
​​​​​​​Month 6	Ascitic fluid	Fluid color	-	-	Yellowish
​​​​​​​Month 6	Ascitic fluid	Total cell count	/µL	-	20
​​​​​​​Month 6	Ascitic fluid	Leukocytes	/µL	-	17
​​​​​​​Month 6	Ascitic fluid	Polynuclear cells	%	-	41.2
​​​​​​​Month 6	Ascitic fluid	Mononuclear cells	%	-	58.8
​​​​​​​Month 6	Ascitic fluid	Erythrocytes	10^3/µL	-	1
​​​​​​​Month 6	Ascitic fluid	Hemoglobin	g/L	-	0
​​​​​​​Month 6	Ascitic fluid	Protein	g/L	-	13
​​​​​​​Month 6	Ascitic fluid	Albumin	g/L	-	11
​​​​​​​Month 6	Ascitic fluid	Bilirubin	µmol/L	-	2.8

Following puncture, abdominal CT was performed and showed residual ascites but no evidence of malignancy or thrombosis (Figures 3-4).

**Figure 3:**
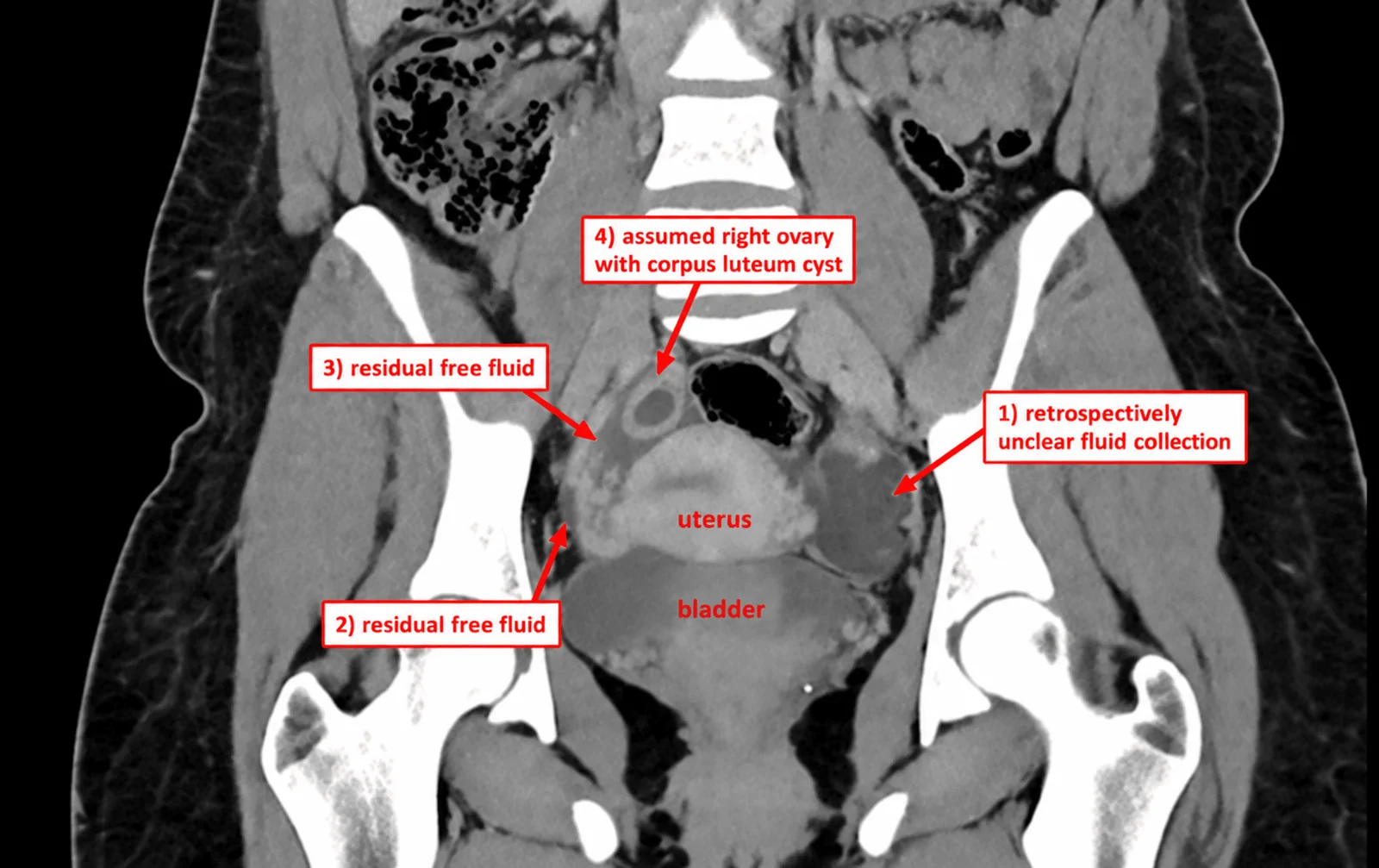
CT of the pelvis with residual ascites

**Figure 4:**
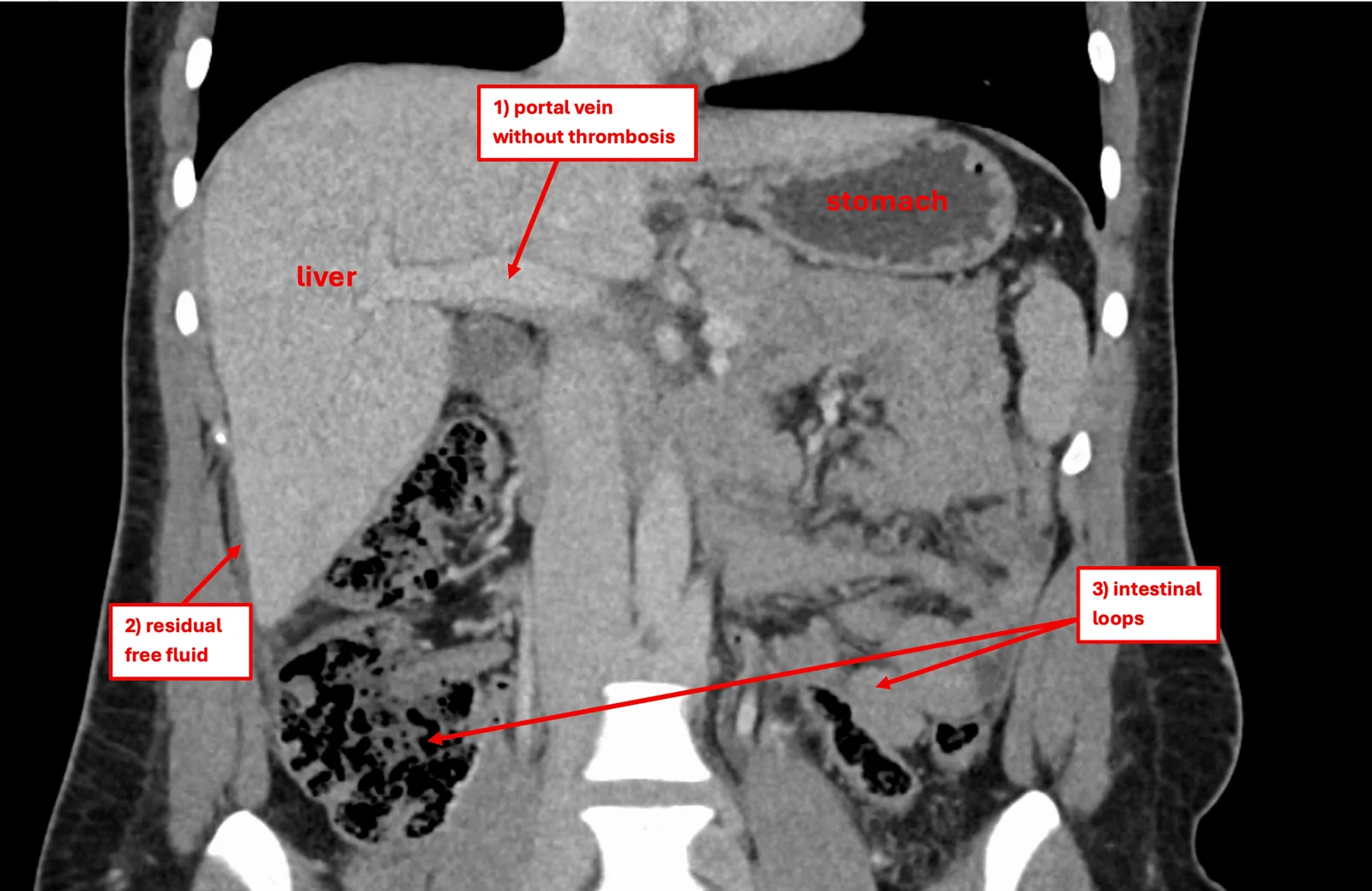
CT of the upper abdomen and liver with no evidence of malignancy or thrombosis

As a portal hypertensive origin was subsequently suspected, a transjugular liver biopsy was performed. Like the preceding investigations, it showed no specific pathological findings (Table 1). During the following months, recurrent intra-abdominal fluid was again noted on follow-up examinations, together with an intrauterine pregnancy. Due to the patient’s distress regarding the unplanned pregnancy and the persistent unexplained intra-abdominal fluid collection, she was referred to the gynecology department for counseling and discussion of a possible pregnancy termination. 

During clinical examination by the gynecology department, a transvaginal ultrasound confirmed an intact intrauterine pregnancy at 7 1/7 weeks of gestation with positive cardiac activity. The right ovary showed a corpus luteum cyst (54.4 x 26.2 x 41.4 mm), most likely functional. Approximately 117 mL of presumed free fluid was also noted, as shown in Figure 5. In retrospect, this most likely represented fluid associated with the cystic lesion rather than true free intraperitoneal fluid. The left ovary appeared unremarkable, measuring 44.3 x 34.2 x 15.0 mm. In hindsight, this likely represented residual, unaffected ovarian tissue, while the massive cystic structure later identified intraoperatively extended far beyond the pelvis and outside the field of view of transvaginal ultrasound, precluding its recognition as arising from the left ovary at this time. In the further course of treatment, medical termination of pregnancy was performed at the patient's request, unrelated to the ongoing diagnostic work-up for the intra-abdominal fluid collection. This part of the clinical course was included in the case report solely to illustrate the chronological sequence of events and was unrelated to the subsequent diagnostic workup.

**Figure 5:**
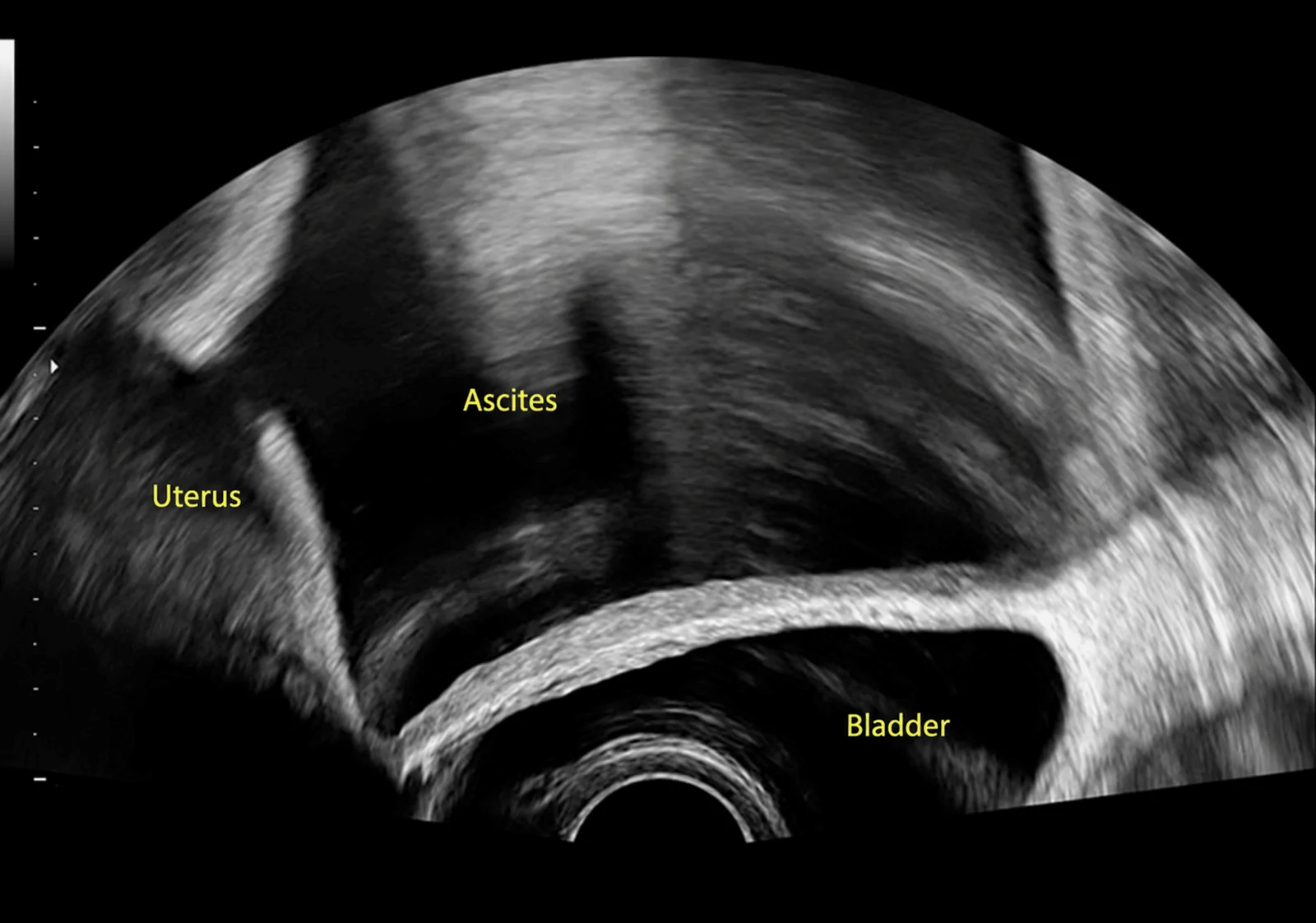
Transvaginal ultrasound showing residual ascites

Follow-up clinical and sonographic assessment by the gastroenterology service again demonstrated an intra-abdominal fluid collection. In contrast to the initial interpretation, the fluid now appeared loculated rather than freely distributed within the abdominal cavity. Again, transabdominal puncture yielded nonspecific findings with a very low cell count (Table 1).

In summary, despite multiple procedures and examinations, the exact cause and nature of the recurrent intra-abdominal fluid collection could not be determined after several months. Neither the post-puncture abdominal CT nor the transvaginal ultrasound was able to demonstrate a distinct cystic lesion. Retrospectively, we therefore assume that the remaining ovarian tissue was already markedly compressed and displaced by the large cystic lesion, making it difficult to identify the ovarian origin of the lesion on imaging. The patient’s case was therefore discussed at an interdisciplinary board and subsequently referred to surgery for further diagnostics using classic diagnostic laparoscopy.

Intraoperatively, a massive left ovarian cyst measuring 18 cm in diameter and containing approximately 2 liters of residual fluid was identified as the presumed source of the symptoms, as shown in Figure 6. The cyst was removed in toto, requiring left oophorectomy. During surgery, the cyst was completely aspirated, as the cytological examination of the aspirated fluid performed preoperatively had shown no evidence of malignant cells, thereby making an underlying malignant process unlikely. Peritoneal surfaces were inspected and considered normal. Oophorectomy was ultimately performed, as no technically feasible alternative for complete resection of the lesion was available intraoperatively. Given the considerable size of the cyst, significant impairment of ovarian function was considered likely. In the context of the patient’s pregnancy during the diagnostic workup and prior fertility counselling by the gynecology team, the decision to perform an oophorectomy was made intraoperatively. The right ovary was also evaluated intraoperatively by the gynecology team, with no evidence of impaired ovarian function. Histopathological examination revealed a serous cystadenoma without evidence of borderline features or malignancy. Peritoneal washing cytology showed reactive mesothelial cells and scant fresh blood components, with no evidence of malignant cells, consistent with the benign histopathological findings. The postoperative course was uneventful, and the patient was discharged in good general condition. Since the operation, the patient has not experienced any recurrence of symptoms during follow-up that led to her presentation in the first place. Ultimately, the findings no longer supported the presence of ascites. Instead, the patient’s symptoms and the intra-abdominal fluid collection were considered to be related to the underlying ovarian cyst.

**Figure 6:**
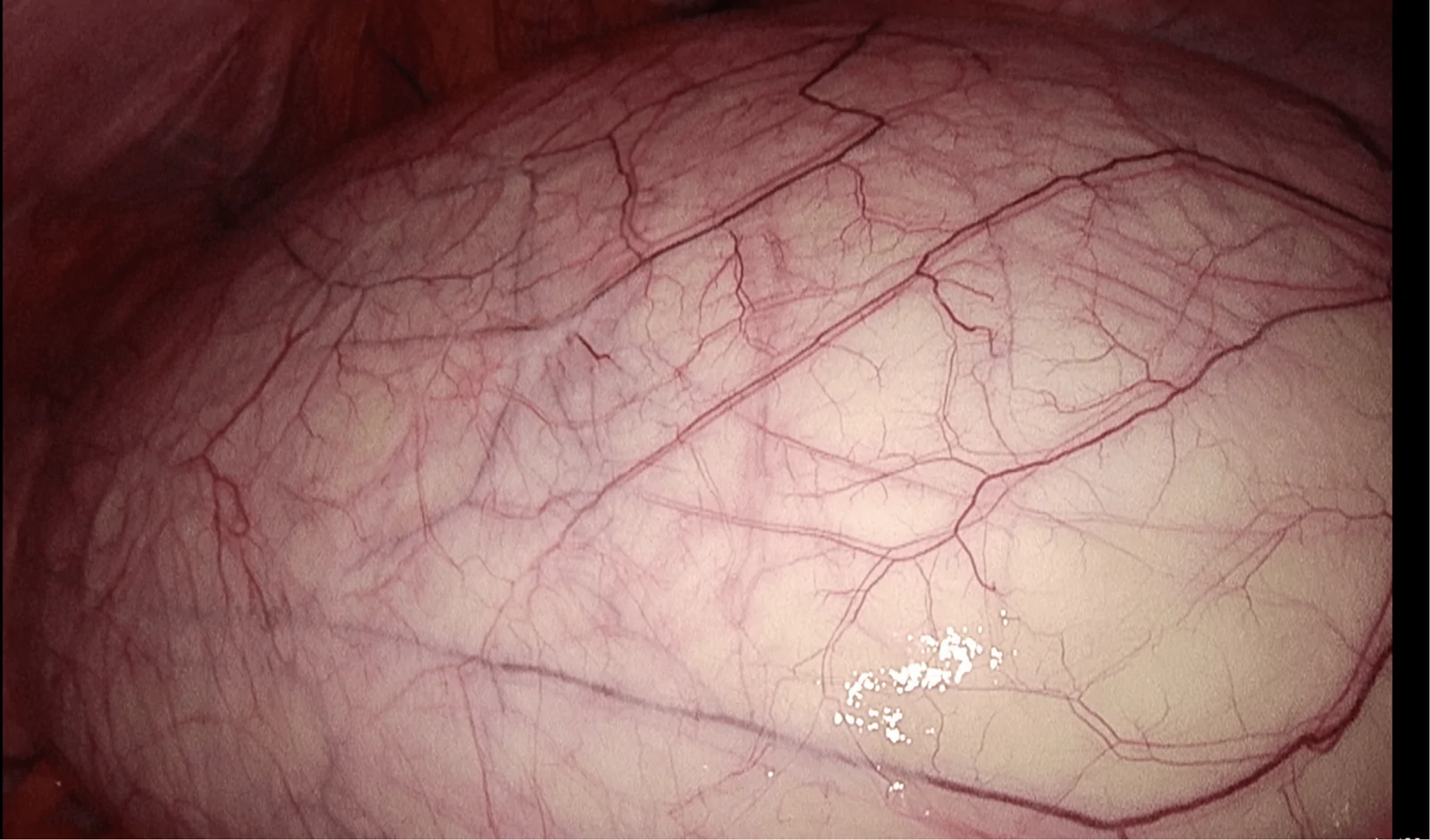
Intraoperative image documentation of the intact ovarian cyst

Table 2 presents the timeline of this case. 

**Table 2:** Timeline of the case

Timeline	Clinical course / Investigation	Key findings	Consequence
Day 0	Initial presentation	Persistent flatulence, progressive abdominal distension, nonspecific abdominal discomfort	Initial imaging performed
Day 20	Presentation and ultrasound via department of gastroenterology	Large amount of intra-abdominal fluid, initially interpreted as ascites, no free floating of bowel loops, no cyst wall present	Further diagnostic workup including bloodwork and ultrasound-guided puncture
Day 25	Paracentesis	Low cell count and no evidence of malignancy or infection	Ascites remained unexplained
Day 25	Laboratory investigations	No evidence of hepatic disease or infection	Further diagnostic evaluation
Day 28	CT of the abdomen and pelvis	Residual ascites but no evidence of malignancy or thrombosis, no cystic lesions	Diagnostic workup continued
Day 33	Transjugular liver biopsy	-	Diagnostic workup continued
Day 55	Reevaluation including ultrasound	Recurrent intra-abdominal fluid, intrauterine pregnancy	Referred to the gynecology department
Day 57	Presentation and transvaginal ultrasound via the department of gynecology	Intact intrauterine pregnancy, corpus luteum cyst within the right ovary, assumed free fluid in the pelvis	Medical termination is planned
Day 60	Medical termination of pregnancy at the patient's request	-	Diagnostic workup continued
Month 6	Follow-up including abdominal ultrasound and paracentesis via the department of gastroenterology	Recurrent intra-abdominal fluid, rather loculated than free, low cell count and no evidence of malignancy or infection	Further diagnostic evaluation with bloodwork
Month 8	Follow-up including abdominal ultrasound via the department of gastroenterology	Recurrent intra-abdominal fluid, rather loculated than free	Referred to the interdisciplinary board and thereafter referred to surgery
Month 8	Evaluation via the department of surgery	-	Scheduled for surgery
Month 8	Laparoscopy was performed	Massive left ovarian cyst	Left oophorectomy
Month 9	Follow-up via the department of gastroenterology	Serous cystadenoma without evidence of malignancy; patient has not experienced any recurrence of symptoms	Further follow-up via a general practitioner

## Discussion

Ascites is a common clinical condition with a broad spectrum of underlying etiologies, most frequently associated with hepatic, cardiac, or malignant disease [1,2]. However, giant ovarian cysts may clinically and sonographically mimic massive ascites, which can lead to diagnostic delay [6]. In the present case, no definitive cause could initially be identified despite repeated punctures, laboratory investigations, cross-sectional imaging, and liver biopsy. The underlying diagnosis was ultimately established by diagnostic laparoscopy, which revealed a giant ovarian cystadenoma. In retrospect, the initial sonographic appearance was therefore most consistent with a large cystic intra-abdominal lesion causing displacement of adjacent structures rather than true free intraperitoneal ascites.

Ovarian cysts are benign and asymptomatic in most cases, but particularly when large, they may present with abdominal distension and may be mistaken for ascites on initial clinical or sonographic assessment [3-5]. Differential diagnosis should especially include benign serous cystadenomas, borderline tumors, and malignant ovarian neoplasms. In rare cases, a so-called pseudo-Meigs syndrome may also be present, in which benign ovarian masses are associated with ascites and occasionally pleural effusions. Clinical presentation is often nonspecific and may range from abdominal distension and pain to gastrointestinal symptoms [10].

In the diagnosis of ovarian cysts, it is of utmost importance to exclude a malignant cause. The most important diagnostic criteria are the patient's medical history, gynecological examination, and ultrasound. Ultrasound is considered an established diagnostic tool because, unlike CT and MRI scans, it is rapidly available and easy to use. However, ultrasound findings alone should be interpreted with caution in borderline cases or when findings are inconclusive. A lack of practical experience on the part of the examiner or limited access to expert interpretation may lead to misinterpretation and consequently to delayed diagnosis. Caution is warranted in cases of unexplained ascites or unclear fluid collections, and additional imaging modalities such as CT or MRI should be considered [6].

Conversely, the risk of malignancy is considerably lower in premenopausal women, such as our patient, which may also explain why tumor marker testing was not prioritized in the initial diagnostic workup. In postmenopausal women, the likelihood of malignancy increases. It is therefore essential to identify features suggestive of malignancy in the medical history, clinical examination, and ultrasound findings, and to refer these patients for further diagnostic evaluation and treatment [3]. In some cases, the additional measurement of tumor markers may be helpful; however, tumor marker levels do not determine the definitive therapeutic approach and are generally of greater importance in follow-up care and surveillance. As the initial working diagnosis in our case was presumed ascites of unclear etiology rather than a cystic ovarian lesion, tumor marker testing was not performed.

Regarding follow-up care, no further surveillance was deemed necessary in our case following definitive treatment of the benign serous cystadenoma. On the other hand, in asymptomatic patients with benign ovarian lesions measuring less than 10 cm that are managed conservatively, regular follow-up ultrasonography is indicated to monitor for changes in lesion size or morphology and to detect any progression at an early stage. In patients with malignant ovarian lesions, gynecologic oncology follow-up is recommended following definitive tumor excision [9,11]. In symptomatic giant ovarian cysts, surgical excision is generally recommended because of diagnostic uncertainty, mass effect, and the need to exclude malignancy [3,7].

In summary, rare gynecological causes and cystic intra-abdominal lesions should be considered in cases of presumed or diagnostically unclear ascites. Diagnostic laparoscopy may play an important role, particularly when noninvasive diagnostic methods fail to establish a definitive diagnosis. Benign ovarian cysts or lesions generally warrant surgical resection only in the presence of symptoms or when they exceed a certain size, with laparoscopy playing a central role in their surgical management. Before diagnostic laparoscopy, relevant contraindications and risk factors should be excluded to ensure patient safety, including severe liver disease, coagulation disorders, and severe cardiopulmonary disease. In some cases, diagnostic laparoscopy may be contraindicated in patients with massive free intraperitoneal fluid, and an open surgical approach should be considered. In cases of suspected or confirmed malignancy, an open surgical approach is generally preferred to facilitate complete resection while minimizing the risk of intraoperative rupture and tumor spillage [7,12]. In our case, laparoscopy was preferred over laparotomy because, at the time of surgery, only a limited amount of free intra-abdominal fluid was present and malignancy was already excluded preoperatively. This approach was associated with a lower risk of intraoperative and postoperative complications, including impaired wound healing and postoperative thromboembolic events [13].

Importantly, in the presence of a cystic lesion of the ovary or another intra-abdominal organ, an underlying malignancy should be carefully excluded before any diagnostic or therapeutic puncture or aspiration is performed, given the potential risk of tumor seeding along the needle tract. The assessment of malignant potential should be based on the lesion’s morphological characteristics on imaging and, where appropriate, supplemented by laboratory investigations, including the determination of relevant tumor markers. In addition, the patient’s medical history and demographic characteristics should be interpreted in conjunction with the clinical presentation and the available diagnostic findings to ensure an appropriate overall assessment of the lesion and its malignant potential.

## Conclusions

This case highlights the limitations of imaging and laboratory diagnostics alone in cases of presumed ascites of unclear origin. Smaller ovarian lesions or atypical findings may be overlooked or misinterpreted on initial imaging studies. In this case, diagnostic laparoscopy proved to be the decisive step in establishing the diagnosis while simultaneously enabling therapeutic intervention. This does not imply that surgical intervention is required in every case of an ovarian cyst. If an ovarian lesion cannot be clearly characterized by the primary imaging modality of choice, transvaginal ultrasonography, further diagnostic evaluation is recommended, depending on the patient’s age and the lesion’s morphology and size. This typically includes MRI, which can often establish a definitive diagnosis. A surgical approach should be considered in patients with symptomatic ovarian lesions as well as in cases where malignancy is suspected or cannot be reliably excluded.

It is also noteworthy that the patient no longer experienced any of the presenting symptoms following surgery. This strongly suggests a causal relationship between the ovarian cyst and the patient’s clinical symptoms, while the apparent ascites was most likely a misinterpreted cystic fluid collection rather than true free intraperitoneal ascites. Given the patient’s young age, otherwise good health, and absence of a relevant family history of malignancy, the initial diagnosis of ascites was considered unlikely. This assessment was further supported by the diagnostic findings, which did not provide evidence of an underlying malignant or other systemic condition typically associated with ascites. The case therefore underlines the importance of interdisciplinary collaboration between internal medicine, gynecology, and surgery in patients with persistently unexplained presumed ascites or recurrent intra-abdominal fluid collections.
